# Orthodontics and Genetics

**DOI:** 10.1590/2177-6709.24.2.092-097.sar

**Published:** 2019

**Authors:** Alexandre R. Vieira

**Affiliations:** 1 Department of Oral Biology, School of Dental Medicine, University of Pittsburgh (Pittsburgh/PA, EUA).

**Keywords:** Genetics, Myosins, Stature, Malocclusion, Genomics

## Abstract

**Introduction:**

Genetics has been suggested as an explanation for the etiology of malocclusions, although some questions, due to the perception that genetic inheritance is tied to a monogenic or Mendelian form of inheritance.

**Objective:**

This paper describes the inheritance of malocclusions, highlighting the areas of knowledge where research has explored mechanisms that explain deviations in patterns of craniofacial growth.

**Conclusion:**

Malocclusions have a complex or multifactorial pattern of inheritance, where more than one gene is involved in the development of the phenotype. There is also the possibility that the environment influences malocclusions.

## Resumo

**Introdução:**

a genética tem sido proposta como uma explicação para a ocorrência das más oclusões, mas isso é questionável, pois a percepção do significado de herança genética está vinculada à herança mendeliana ou monogênica.

**Objetivo:**

o presente artigo visa discorrer sobre a herança das más oclusões e ressaltar as áreas do conhecimento nas quais a pesquisa tem explorado mecanismos que explicam a ocorrência de desvios do padrão de crescimento facial.

**Conclusão:**

as más oclusões têm um padrão de herança complexo ou multifatorial, no qual mais de um gene está envolvido no desenvolvimento do fenótipo. Isso quer dizer que existe, também, uma potencial influência do ambiente nas más oclusões.

## INTRODUCTION

Estimations of the frequency of malocclusions exist for many countries, and in general they are high, with approximately one third of the population needing treatment.^1^

Malocclusion is not a disease, but a condition defined as a series of deviations that in some cases impact quality of life. There is no evidence that orthodontic treatment improves oral health or function, but the treatment is justified by the potential social and psychological improvement that a change in appearance can bring.^2^

There is interest in understanding how malocclusions develop. Many investigators approach the question exploring a mechanistic hypothesis. Defining growth trajectories may help understand expected patterns, but does not provide an explanation for why such events occur. Exploring individual susceptibility to malocclusion will allow for determining why some individuals have more deviations in craniofacial growth. In this paper, inheritance patterns of malocclusion will be discussed.

## MALOCCLUSIONS HAVE MULTIFACTORIAL INHERITANCE

**Figure 1:**
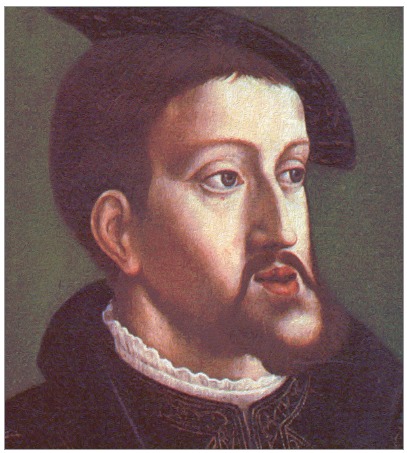
Profile view of Carlos V of Spain and Germany at 17 years of age. His family included 13 lineages of European royalty and 409 documented individuals,^33^ with 321 with mandibular prognathism varying from mild to severe. Analyses of this family suggested that mandibular prognathism has an autosomal dominant mode of inheritance, and cases that did not fit well may be due to consanguinity. In some cases, the prognathism escaped a generation and penetrance was estimated at 0.88.

The suggestion that malocclusion has a genetic component comes from observations of mandibular prognathism (frequently associated with Angle’s Class III) segregating in families. Probably the best-known example is the House of Habsburg, which produced emperors and kings of Bohemia (current Czech Republic), England, Germany, Hungary, Croatia, Illyria (a region of Austria), the Mexican second empire, Ireland, Portugal, Spain, and several administrators and principalities of Denmark and Italy (Fig 1).^3^ Since many cases of mandibular prognathism aggregate in families, there is the perception that it follows an autosomal dominant Mendelian mode of inheritance (monogenic or single gene). The perception that one gene with a main effect leads to mandibular prognathism^4^ motivated linkage^5^ ^-^ ^8^ and association^9-17^ studies under the hypothesis that a strong genetic effect can be identified even with relatively small sample sizes (definitions of linkage and association studies are provided at the end of this article). These results are inconsistent, suggesting that monogenic inheritance and a gene with a major effect are not the best explanation for the majority of cases of malocclusion. Currently, it is understood that inheritance of mandibular prognathism and malocclusions in general is multifactorial or complex, which means that more than one gene (instead of just one) contribute to the establishment of malocclusion, and these genes can be influenced by the environment. Like for other conditions, there are exceptions, and a major gene effect with autosomal dominant inheritance may be possible.

## AN UNCONVENTIONAL MYOSIN

Knowing that malocclusion is influenced by more than one gene and that these cases are clinically heterogeneous, it was first proposed to approach the question by studying clinically well-characterized cases^18^. Profile photos were obtained from all study participants, showing soft tissue relationships (concave or convex) and cephalometric measurements, to classify individuals in orthognathic versus prognathic. More specifically, it was focused on measurements of Steiner, ANB, Wits and the Downs A-B plane. According to the Steiner analysis, the ANB angle smaller than 2 degrees indicates that the mandible is positioned ahead of the maxilla. Were evaluated the individuals with ANB values smaller than 2 degrees to determine if the discrepancy is due to the maxilla being smaller than average. Such cases were not considered true prognathic individuals, but cases with a normal size mandible apparently protruded due to anteroposterior maxillary deficiency. The Wits values were also assessed, which indicates anteroposterior relationships according to intracranial references. A negative Wits value indicates a skeletal Class III, and the lower the Wits value, the more severe the case of Class III. A Downs A-B plane with an angle of 4.6 or higher indicates a skeletal Class III, although this measurement is more severe when the individual has a more accentuated pogonium. Additional clinical criteria for Class III were included, such as Class III relationships of molars and canines, and negative overjet.

In a study with north-American families of Hispanic origin that showed an autosomal dominant pattern of mandibular prognathism, five *loci* (chromosomal regions) were identified as being linked to mandibular prognathism due to maxillary deficiency: 1p22.1, 3q26.2, 11q22, 12q13.13, and 12q23^19^ [each chromosome has a short (“p” for “petit”) and a long arm (“q” for “queue”), and each arm is divided into cytogenetic bands, which are called p1, p2, p3, q1, q2, q3 etc., counted from the centromere to the telomere]. When these five regions were studied, it was found an association with MYO1H in 12q23 in north-Americans.^18^ MYO1H is a unconventional myosin and the present results were independently replicated in a group of Brazilian patients with prognathism without maxillary discrepancy,^20^ and in prognathic individuals from Midwestern regions in the United States.^21^ The mutation of a proline to a leucine in the position 1001 of the MYO1H protein can be a functional variant in humans and orthologs (similar DNA sequence in distinct species, suggesting they had a common ancestor) of myo1h in zebrafish (*Danio rerio*) are expressed in the mandible,^22^ suggesting a function during development. This accumulated evidence suggests that MYO1H may be a predictor for the establishment of prognathism, and may help in determining which patients respond better to treatment. 

## SPRINTERS VERSUS MARATHON RUNNERS

**Figure 2:**
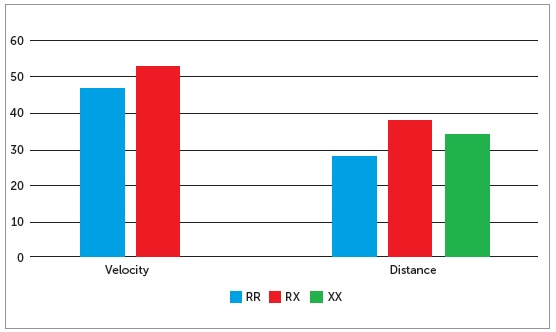
Frequency of ACTN3 R577X genotypes in track and field Olympic athletes that are sprinters versus long distance runners.^23^ The XX genotype is more common in long distance runners. The frequency of X is also more common in Class II individuals and less common in individuals with deep bite.^24^

The idea that craniofacial deformities and malocclusions can be influenced by factors not directly related to the skeletal basis in intriguing. Motivated by the results with MYO1H, genes that code for skeletal muscle alpha-actin were tested: ACTN2, which is expressed in all muscle fibers, and ACTN3, which is expressed in fast-twitch fibers (type 2). The frequency of a genetic variant in particular, the mutation R577X, is increased in people who run longer distances, and decreased in sprint runners (Fig 2).^23^ When associations were tested for genetic variation in the genes that code for alpha-actin and sagittal and vertical definitions of malocclusion, it was found that skeletal Class II individuals more frequently had two copies of 577X and less number of type 2 fast-twitch muscle fibers in the masseter.^24^ This evidence suggests that the function of the connective tissue, in particular muscles, has a role in the establishment and severity of skeletal deformities. 

## FACIAL ASYMMETRY

A perception of symmetry between the two sides of the face defines attractiveness. Deviations of this harmony, which are referred to as asymmetry, bring discomfort and low self-esteem. In general, the right and left sides of the face mirror each other, and keeping symmetry is apparently important for midline definition. The lefty proteins are responsible for interrupting body symmetry to allow the normal positioning of the heart, lungs, and stomach.^25^ In the face, a similar event of expression of lefty occurs on the left side only, which has not been identified on the right side,^26^ and this difference may explain, at least in part, why clefts affect the lip twice as much on the left side.^27^ Similarly, facial asymmetry is typically found on the left side.^28^

**Figure 3:**
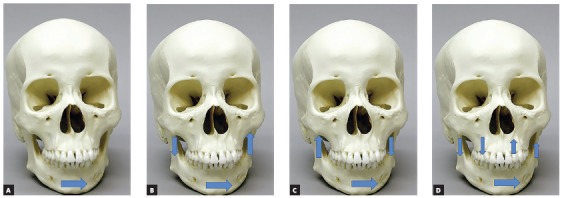
Asymmetries studied by Chung et al.^29^: A) asymmetry of the body of the mandible, B) asymmetry of the ramus of the mandible, C) atypical asymmetry, and (D) C-shaped asymmetry.

When individuals who have undergone orthognathic surgery to correct craniofacial deformities were studied, four types of asymmetry were detected: asymmetry of the body of the mandible, asymmetry of the ramus of the mandible, atypical asymmetry, and C-shaped asymmetry (Fig 3).^29^ Genetic variation in ESR1 and *ENPP1*, which are genes involved in bone mineralization and that were associated with Class II and Class III, respectively,^30^ may influence facial formation in cases of asymmetry.^29^ ENPP1 is also associated with mandibular condyle shape variation.^31^ Individuals with asymmetry of the body of the mandible more often showed genetic variation in ENPP1, when compared to other types of asymmetry (Fig 3). People with atypical asymmetries or C-shaped asymmetry more often had variation in ESR1. Maybe, what is most relevant is that only 3% of the cases considered symmetrical had temporomandibular joint disorder, in comparison to 78% of people with asymmetries described in Figure 3. The challenge continues to identify which individuals benefit from orthognathic surgery. About 7% of patients end with their temporomandibular joint dysfunction worsened after orthognathic surgery, and most of them were individuals without asymmetry.

## ORTHODONTIC TOOTH MOVEMENT

The initial response to the compressive forces involve activation of genes that control angiogenesis, inflammation, osteoblast formation, and extracelular matrix remodeling.^32^ The osteopontine protein is thought to be a potential biomarker to predict the result of orthodontic treatment, due to its role in bone and periodontal remodeling.^33^ This idea can be proposed to all aspects discussed thus far. Genetic variation, or variation in the control of gene expression may help predict the results of treatment and undesirable consequences, such as temporomandibular joint dysfunction. Research in Orthodontics, exploring individual susceptibility, and the combination of technology that allows exploring genomic and epigenomic roles may help to determine the function of the connective tissue on the establishment of the skeletal basis of the face, and to anticipate the results of interventions in the patterns of growth. More specific cases, such as the amount of external root resorption secondary to orthodontic tooth movement and variation in the speed each patient supports orthodontic tooth movement without negative consequences are also the focus of genetic evaluations in the future.

## FINAL CONSIDERATION

Genetics explains a great deal of variation seen in the population when facial deformities and malocclusions are considered. However, genetics is not synonymous of a deterministic concept in which a single gene, segregating in families, determines malocclusion. These monogenic models explain very few cases of malocclusion and the other human diseases, as well as traits such as height, weight, amount of sugar in the circulating blood, blood pressure, intelligence, behavior, and sexual orientation. All these traits, as well as the majority of human diseases and congenital defects, have complex or multifactorial modes of inheritance, which can be influenced by the environment, and determine the presence of the majority of traits and diseases. 

## Glossary

» Association: Observational study that tests in individuals if a particular genetic variant is more frequent than another one, depending on the person being affected by the disease or being a carrier of a trait of interest.
» Linkage: The tendency that genes and other genetic markers are inherited together, since they are physically close on the same chromosome.
